# A pilot protocol for surveillance of infection and antibiotic prescribing in primary healthcare across the globe: Antibiotic Prescribing in Primary Healthcare Point Prevalence Survey (APC-PPS)

**DOI:** 10.12688/wellcomeopenres.23420.1

**Published:** 2025-01-21

**Authors:** Aislinn Cook, Jan Goelen, Catrin E. Moore, Jennifer Martin, Koen B. Pouwels, Michael Sharland

**Affiliations:** 1 Centre for Neonatal and Paediatric Infection, Institute of Infection and Immunity, School of Health & Medical Sciences, City St George’s, University of London, London, England, UK; 2 Health Economics Research Centre, University of Oxford Nuffield Department of Population Health, Oxford, England, UK

**Keywords:** Primary Care, Antibiotic prescribing, Infections, AWaRe system, Point prevalence survey (PPS), Antibiotic use, Low and middle income countries (LMIC)

## Abstract

Little data is available from the primary healthcare setting in low- and middle-income countries to describe the burden of clinical infections and antibiotic prescribing proportions for those infections. The AWaRe Antibiotic Book provides a framework for assessing antibiotic prescribing in primary healthcare but requires understanding both frequency of clinical infections and their antibiotic prescribing proportions. The Antibiotic Prescribing in Primary Healthcare Point Prevalence Survey (APC-PPS) project is a series of point prevalence surveys conducted at primary healthcare facilities in LMICs to capture the frequency of consultation for different clinical infections and diagnoses and the frequency and type of antibiotic prescribing associated with these infections in primary healthcare facilities.

This study aims to assess the feasibility of using a PPS methodology to collect data on clinical presentation and antibiotic prescribing in primary healthcare settings. The data collected are necessary to be able to summarise relative rates of presentation of different clinical infections and antibiotic prescribing practices to inform global estimates of antibiotic use and inform the development of surveillance methods and representative sampling frames.

Each site will conduct 6-8 point prevalence surveys over the course of 12 months. Completely anonymous data on age, sex, relevant comorbidities, infection symptoms and diagnoses and antibiotic prescription are collected for patients of all ages with acute infection symptoms (up to 14 days of symptoms) who present to the facility on the day of the survey. No identifiable data will be collected from individuals. Data is collected via ODK Collect and stored in a secure ODK Cloud server hosted by City St. George’s, University of London. Sites will be active between early 2023- end 2024, with regular interim data analysis scheduled and final data analysis planned by mid 2025. All required local and national ethical and regulatory approvals will be obtained prior to sites starting.

## Plain Language Summary

The Antibiotic Prescribing in Primary Healthcare Point Prevalence Survey (APC-PPS) study aims to fill a data gap in low and middle income countries to better understand the frequency that patients present to primary healthcare facilities with different types of infections and to understand which patients receive an antibiotic prescription and which antibiotic. Antibiotic prescribing patterns will be compared against antibiotic prescribing guidelines such as the AWaRe Antibiotic Book to understand how prescribing patterns follow recommended guidelines. The data are captured using an easy-to-use mobile app (ODK) with offline capabilities. The data from this study will also be used to understand the feasibility of using this type of methodology and data collection app in different primary healthcare settings and different countries to inform the development of a standard representative methodology for capturing these types of data. The study has been implemented in 66 sites in 9 countries (6 in Africa and 3 in Asia) between April 2023 - November 2024. There are a diverse types of primary healthcare facilities participating in the study including outpatient departments, community dispensaries, community health centres, polyclinics and more.

## 5.1 Introduction

The Antibiotic Data to Inform Local Action (ADILA) project is a Wellcome Trust funded study that aims to use data on antimicrobial consumption, resistance, and clinical outcomes to derive novel frameworks that can inform the development of national and local policies to improve antibiotic prescribing. One of the key objectives focuses on developing a primary care clinical antibiotic prescribing framework that integrates clinical infection presentation/diagnoses with antibiotic prescribing.

The World Health Organization (WHO) released the WHO Essential Medicines List (EML) AWaRe Antibiotic Book in 2022 ^1, 2^, providing detailed guidance on the choice of antibiotic drug, dose and duration for 35 common infections in adults and children in primary care and hospital settings. The AWaRe Book is built around the AWaRe system (Access, Watch, Reserve), a classification for antibiotics related to their potential for selecting for resistance. Access antibiotics are the first choice for the most common infections and should be widely available and generally have a lower potential for selecting resistance; Watch antibiotics are broader spectrum antibiotics with higher potential for resistance so should be used only for specific indications; and Reserve antibiotics are important last line antibiotics, reserved for the management of multidrug-resistant pathogens.

The AWaRe Antibiotic Book recommends that 9 out of the 10 most common infections in primary care should be treated with Access antibiotics. The book also highlights that low-risk patients with mild infections may not need antibiotic treatment. There is very little data from the low and middle income (LMIC) setting describing the variation in the relative incidence of clinical infections presenting to different types of primary care/ambulatory care facilities.

Point prevalence surveys (PPS) are a simple method to measure antibiotic use (or other medicines use). They are generally implemented on a single day and capture anonymous data on patients receiving an antimicrobial on the given day including demographics, specific antibiotic and indication. These types of surveys have been used successfully globally to measure antibiotic use and indication for prescription in hospitals ^3– 14^ and the WHO has a published methodology ^3^ for conducting these types of surveys in the hospital setting (Global Antimicrobial Resistance and Use Surveillance System, GLASS). These types of point prevalence surveys have not been widely adapted to capture antibiotic use in the primary healthcare setting ^15^ despite majority of antibiotic prescribing occurring in these settings compared to hospitals.

To inform and monitor local and national antibiotic use targets and quality indicators, understand the feasibility of collecting basic clinical and prescribing data in the primary healthcare settings using basic tools such as point prevalence surveys is necessary. This includes the sampling frame, costs and the minimum data required to provide reasonable levels of precision of estimates of antibiotic use accounting for variable clinical burden to allow comparison of actual use compared to local and global guidelines.

Very little data is available from the primary healthcare setting in LMICs to describe the burden of clinical infections and antibiotic prescribing proportions for those infections. The few datasets available include data on (estimates of) infection and incidence by country alone (e.g. Global Burden of Disease (GBD); https://www.healthdata.org/), antibiotic prescriptions sometimes linked to diagnosis ^16^ or sales data without information on linked diagnoses (e.g. IQVIA MIDAS database, WHO GLASS Antimicrobial Consumption (AMC) module). However, there is currently insufficient data to compare observed prescribing patterns for common infections compared to local, national or WHO guidance. 

This project aims to understand the feasibility of using PPS methodology to collect data on patterns of clinical presentation and antibiotic prescribing / dispensing in primary healthcare.

The project also aims to determine the frequency of consultation for different clinical infection presentations/diagnoses together with the frequency of those prescribed and not prescribed and type of antibiotic prescribing (if prescribed) for these infections in primary healthcare facilities. 

## 5.2 Objectives

1. Quantify the frequency of people presenting to primary healthcare facilities with an infection and the relative frequency of presentation of different clinical infections.
2. Quantify the proportion of those presenting with clinical infections that receive an antibiotic prescription
3. Of those who receive an antibiotic prescription, quantify the proportion of each AWaRe antibiotic prescribed
4. Inform the design of a future optimal sampling strategy to obtain a representative sample of sites within a region or country.

## 5.3 Outcome

To determine the feasibility of using PPS methodology for surveillance of antibiotic use and to inform the sampling strategy of future surveillance surveys.

To understand the relative presentation rates of clinical infections covered in the WHO EML AWaRe Antibiotic Book in different settings and to understand antibiotic prescribing rates for these infections.

Local data summaries and graphics will be shared with sites to inform local initiatives.

## 5.4 Study design

### 5.4.1 Study design and setting

The Antibiotics in Primary Care-Point Prevalence Survey (APC-PPS) is a multi-centre, multi-country series of point prevalence surveys (PPS) conducted in a range of primary healthcare settings that prescribe or dispense antibiotics (e.g., primary care facilities, community health centres, hospital outpatients/ambulatory care, pharmacies, etc.). Sites participating are from Tanzania, Ghana, Malawi, Kenya, South Africa, Namibia, Pakistan, Bangladesh and Thailand. Participating countries were identified through stakeholder engagements through existing networks.

Study set up commenced in Autumn 2022 with outreach to country and site partners together with any required local ethical approvals. The first site opened in early 2023 with additional sites opening throughout 2023 and 2024 (dependent on local ethics timelines). Each site will conduct multiple PPS over 6 months from the first survey. The latest any site will be able to open in this phase is mid 2024 to allow for all data collection to be completed by the end of 2024. Full analyses will be completed 6 months after the last site finishes data collection. Interim analyses have been conducted at the end of 2023 and in 2024.

**Figure 1.**
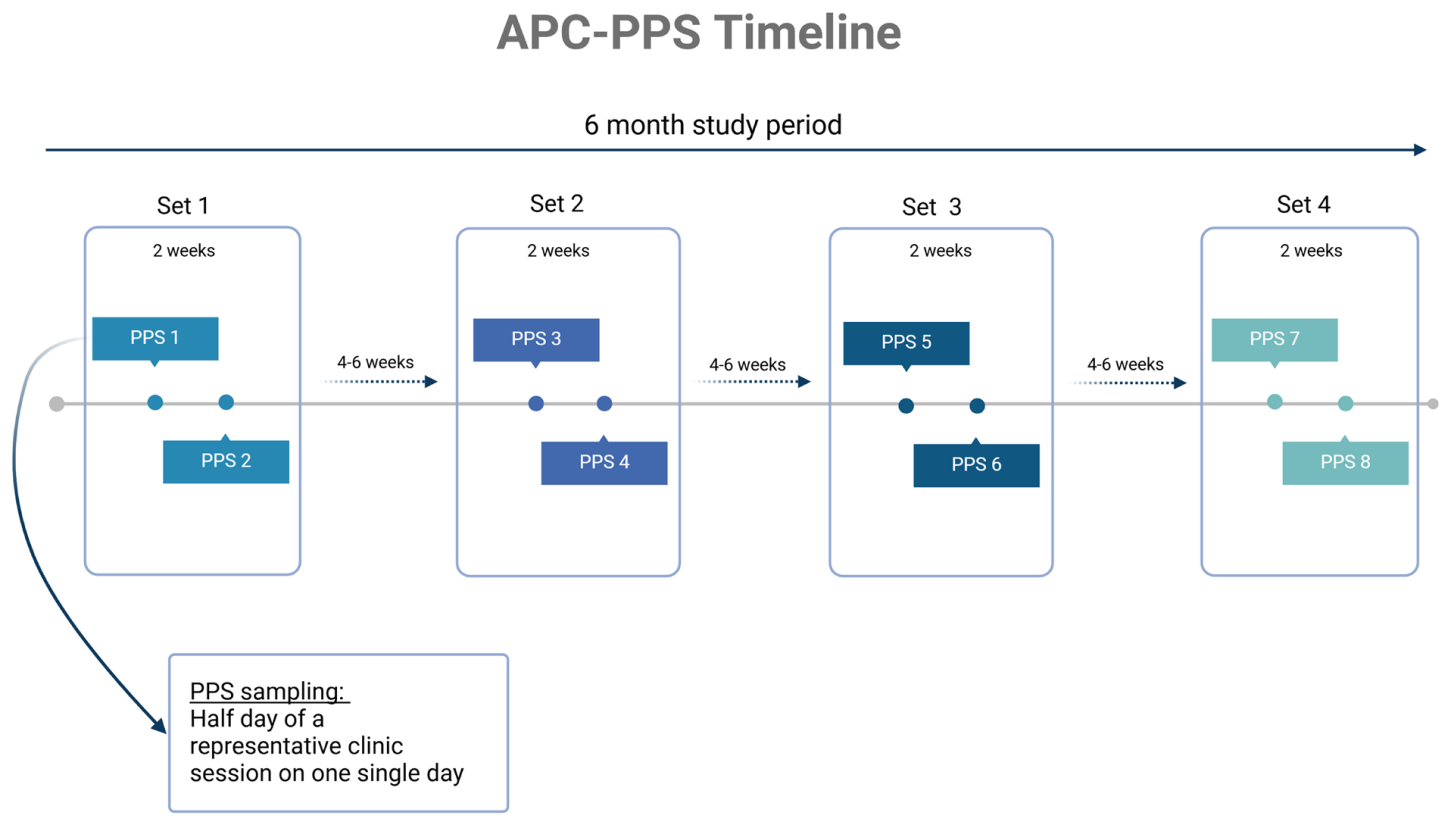
Study schematic timeline of APC-PPS for each site. Created with BioRender.com.

Each site will collect data over the course of an approximate 6-month period to capture any seasonal differences in infection burden or antibiotic prescribing. Sites will conduct two half-day surveys in a two-week period (a “set” of surveys) and repeat these sets every 4–6 weeks conducting where possible a total of six to eight surveys in 6 months ( Figure 1). All clinical decisions are determined by local staff at the point of care; this observational project is only collecting information on the prescribing decisions made.

### 5.4.2 Sample recruitment

***5.4.2.1 Country eligibility***

Any country is eligible to participate in the point prevalence survey provided a country coordinator has been identified and the appropriate regulatory and ethical obligations are met.

***5.4.2.2 Facility/site eligibility***

Any facility within a country providing primary healthcare with staff (e.g. physician/doctor, medical officer, nurse, pharmacist, pharmacy technician, etc.) who can evaluate patient symptoms and then prescribe or dispense antibiotics are eligible to participate provided the site has identified a site point-of-contact and has met any regulatory and ethical obligations required. Sites will also need to ensure teams collecting data have access to Android phones or tablets to use the Open Data Kit (ODK) Collect mobile app.

***5.4.2.3 Patient eligibility criteria***

This study will collect anonymous data on all eligible patients presenting to participating facilities on the day of the survey with acute infection symptoms. Data from each site will be grouped by a facility identifier to allow stratification by facility factors within a single country in order to assess heterogeneity.

**Inclusion criteria**

All children and adults presenting with acute infection symptoms on the day of the survey should be included. Acute is defined as symptom(s) occurring for less than 14 days.

Eligibility of patients presenting to the facility meeting these criteria will be determined by the local site team using the infection symptoms/diagnoses of interest outlined in Table 1 as a guide. Local clinical discretion will be used to determine patients with acute infection symptoms. Patients with underlying chronic conditions presenting because they have acute infection symptoms should be included.

**Table 1.** List of presenting infection symptoms / diagnosis and additional symptoms questions for each infection presentation.

Presenting infection symptoms / diagnosis	Additional symptoms queried
*•* Does the patient have / report having a fever?	O Persistent fever lasting 7 days or longer O Suspected enteric fever O Patient received anti-malarial prescription for this fever episode
*•* Acute cough	O Cough >5 days O Shortness of breath/ difficulty breathing O Chest pain
*•* Sore throat/ pharyngitis/ tonsillitis	
*•* Facial pain or pressure/ sinusitis	
*•* Runny nose/ nasal congestion / coryza	
*•* Ear pain/ acute otitis media	O Uni-lateral ear pain O Bi-lateral ear pain O Otorrhoea/ ear discharge
*•* Toothache/ tooth abscess	
*•* Acute diarrhoea / gastroenteritis	O Bloody diarrhoea
*•* Increased urgency or frequency of urination / urinary tract infection (UTI)	O Blood in urine
*•* Painful urination	
*•* Genital discharge / sexually transmitted infection (STI)	
*•* Wound/ burn/ bite infection	
*•* Skin rash / spots – without swelling	
*•* Skin swelling / redness / warmth / pain	O Swollen lymph nodes
*•* Other primary presentation/ diagnosis	Please specify other symptoms/ diagnosis:

**Exclusion criteria**

Patients presenting to the facility seeking care for underlying chronic conditions as their primary reason for consultation will be excluded. Patients meeting the exclusion criteria will be determined by local clinical teams. Patients with chronic conditions (eg chronic obstructive pulmonary disease) who present with acute signs of infections should **not** be excluded.

### 5.4.3 Sampling strategies

***5.4.3.1 Sampling strategy for site selection***

Sites will be selected as a convenience sample based on the availability of a site team able to conduct data collection activities and for which they have met any local and/or national regulatory and ethics requirements.

As this study aims to determine the variability of clinical presentation and associated antibiotic prescribing rates there is no minimum number of sites per country defined. We aim to include a mix of private and public facilities and a mix of different types of outpatient and primary healthcare facilities and urban and rural facilities.

***5.4.3.2 Sampling strategy for point prevalence surveys***

Each primary care facility will conduct 2 single-day point prevalence surveys in a two-week period (a “set”) and repeat this every 4–6 weeks for 6 months in total. Each survey will be conducted for a half a day or approximately 4 hours of a ***representative*** standard clinic session on a single day. Surveys will be conducted over 6 months to capture any seasonal variation in clinical infections presenting to primary care ( Figure 1). Sites were advised to avoid conducting the surveys on days of any specialist clinic sessions that may influence rates of presentation. 

*Number of patients per facility:*

As this is a point prevalence survey conducted within a specific period of time at different facilities, it is not possible to identify the specific size of the sample needed for each facility. The size of the facility and local population will influence the number of patients attending.

As presentation rates of different clinical infections will likely vary by country or facility it is difficult to determine the specific sample size of each type of infection at each facility. Some conditions are rarer than others and may not be captured on the specific day of the survey at each facility. In order to try to capture some of this variability in presentation we will conduct surveys in two half-days across different days in a two-week period.

This study will provide data on the number of patients with each diagnosis per time unit which will help to determine future sample size calculation for subsequent study designs aiming to define regional or country-level representativeness.

### 5.4.4 Consent

Data collected for this study will be fully anonymised at the time of data capture and will not contain any personal or identifiable information and clinics are only identified by a facility ID code in the data. In many settings, these types of data would be routinely collected as part of a clinical audit to assess adherence to local guidelines so we will not be obtaining individual informed consent from the patients for the purposes of this study. This is also to ensure feasibility by reducing the disruption to clinics and routine practice as formal written consent is more time burdensome and disruptive to routine practice. To understand prescribing practices in primary care, it is essential to capture as many patients presenting as possible to understand the true rates of different infections and true prescribing rates for each type of infection; therefore reducing burden on sites and disruption to patient visits was essential to ensure representative sampling.

## 5.5 Data collection

### 5.5.1 Data collection platform

Data will be collected and managed using Open Data Kit (ODK; https://getodk.org/). ODK allows for offline data collection through a mobile app, ODK Collect, which improves the ease of implementation in global sites.

Data will be hosted using ODK Cloud, the hosting service provided by ODK. Data will be hosted on a private server, and only authorised project users will have access to the database. ODK Cloud is based in a secure GDPR-compliant EU data centre, all data is encrypted in transit and at rest in the ODK Cloud, and the database and data are backed up continuously.

Site data collectors will collect data using the ODK Collect app, an open-source Android application that supports offline data collection. Users will be allocated to country-specific forms using a site-specific QR code. If QR scanning does not work at a site, users can connect to the project on their computers or mobile phones using a URL and username/password. Data is synced automatically from the app to the central server when the device is connected to the internet/mobile network. Users can also submit records when they are not connected to the internet/mobile network, all data will be synced with the central server when the device is next connected to the internet/mobile network.

ODK Collect allows for an anonymous device-ID to be collected to distinguish users at each site assuming each data collector uses a unique device. In cases where one device may be used by multiple data collectors, data collectors were able to login with their unique QR code to distinguish user data collection when needed. To understand the feasibility of data collection of this type, the amount of time it takes for each record to be collected will be captured using ODK Collect app’s metadata features. This will capture the elapsed time from when the data collector started data entry for that record to when they submitted that record.

Data in the ODK Cloud server will only be accessible to the core project team based at City St George’s University of London (CSG). Sites will not have access to view or edit their data or data from other sites after it has been submitted to the central server from the mobile app (ODK Collect). At the end of the data collection, each site will receive a copy of their raw data in CSV format. This will be sent from the core project team at CSG via CSG iDrop, a secure, encrypted, file transfer system. Sites will also receive summaries of their data which is outlined further in the data sharing and publication policy (available in extended data repository). 

***5.5.1.1 Training***

Country coordinators will be trained in the protocol and data collection processes by the central Project Team at CSG. Site leaders will be trained by both the country coordinators and through online videos and documents. Data collectors at each site will be trained in ODK Collect and to understand the inclusion criteria by site leaders and online videos produced by the central Project Team. We utilised a cascading training model where country and site leadswere trained during live video training by the central Project Team at CSG and they were responsible for providing appropriate training of site data collection teams to adequately implement the project. Site leads were provided training slides and additional training documents and all teams had access to the study-specific online training platform hosted on The Global Health Network ( APC-PPS • Antimicrobial Resistance (AMR) (tghn.org)). Training materials are available in the extended data repository.

### 5.5.2 Data collection workflow 

***5.5.2.1 Facility-level data***

On the day of each PPS, data collectors will complete a form about the facility and the PPS time slot including information on total presenting patients, data collection methods and antibiotic availability (if applicable) on site on the day of the survey. Once over the time period, the site will complete an overall facility information form providing information on facility characteristics and an depth antibiotic availability and pricing form. The data collected in these forms is described in Table 2. The full data collection forms are available in the extended data repository.

**Table 2.** Data collected in the facility form and PPS time slot.

Overall facility information	PPS-specific information
*•* Facility name *•* Facility location (city, country) *•* Type of primary healthcare facility *•* Public (e.g. government) or private *•* How patients pay for visits and medications *•* If there is a pharmacy onsite ○ List of antibiotics available at the facility ○ Cost of antibiotics available	*•* Date of survey *•* Time slot for the PPS (e.g. start and end time indicating morning, afternoon or evening) *•* Total number of attenders (for all conditions) on the day of the PPS – split by number of adults (≥18 years) and children/ babies (<18 years) *•* Qualification(s) of prescribers/dispensers on day of survey (e.g. nurse, doctor, pharmacist, medical officer, etc) *•* Number of prescribers/dispensers during the survey time period *•* Antibiotics in stock on day of survey (if pharmacy is onsite)

***5.5.2.2 Patient-level data***

Fully anonymous data will be collected on the day of the PPS for all patients who present with acute (present for <14 days) infection symptoms. Each record (patient encounter) collected is linked with a site/facility identifier based on the QR code/login used by each user at that facility which will enable analysis to account for facility differences in prescribing. The data collector will record the necessary clinical data about the patient's presentation (including patient / parent (or guardian) reported symptoms) and whether they were prescribed an antibiotic at this visit (as described in section 5.5.2.3).

**Figure 2.**
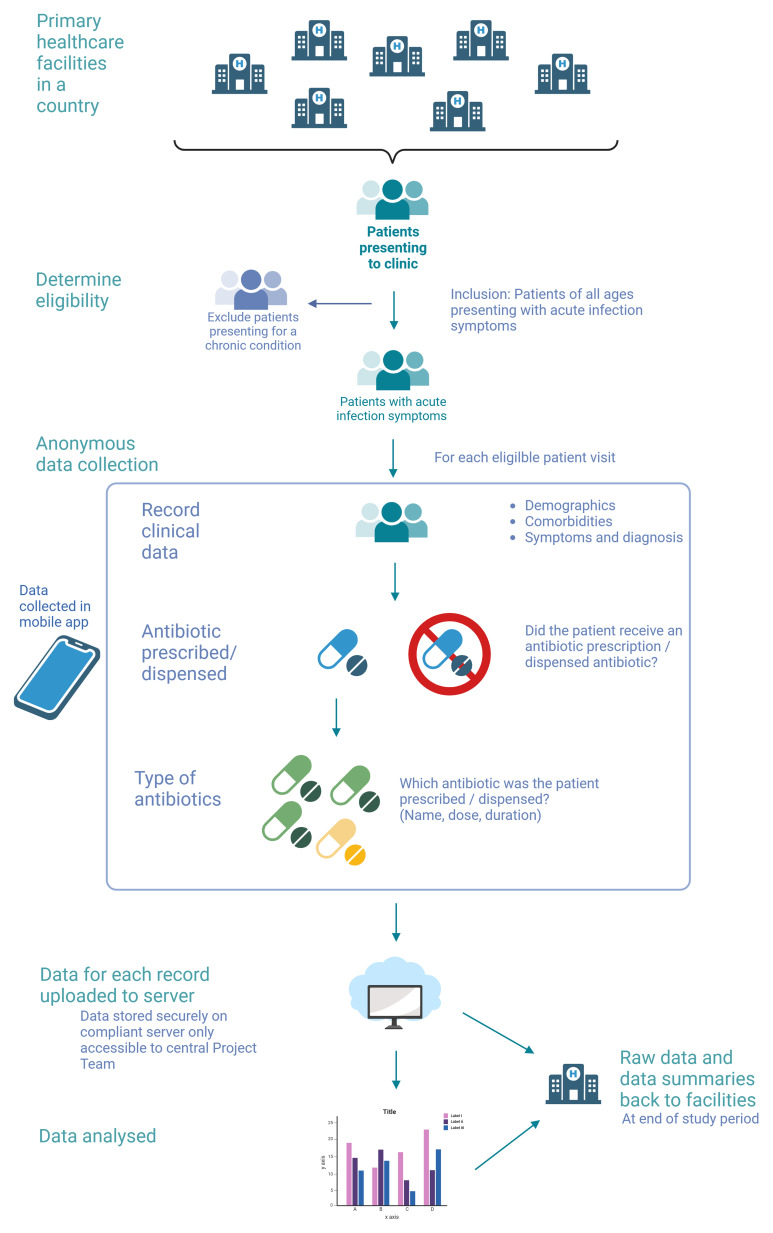
Data collection workflow diagram for the APC-PPS study. (created with BioRender.com).

Once data have been entered on the form in the ODK Collect app, the data collector will save/submit the finalised form at which point ODK will generate a unique ID number for that record. If the mobile device has internet access at the time, the data will be automatically uploaded to the server. If the mobile device is not connected to the internet, all data will be uploaded to the server when the device is next connected. Figure 2 illustrates the data collection flow.

***5.5.2.3 Consultation data collected***

No personal or identifiable data about patients will be collected on the case report forms (CRFs). Age (years for patients ≥2 years; months for <2 years) and sex of the patient will be collected to allow for reporting clinical presentation and prescribing rates adjusted for different patient populations. Certain diagnoses are more common in children (e.g. ear infections) or in women (e.g. urinary tract infection, UTI) so these data are necessary to appropriately adjust to wider populations. These data are essential to interpret the presentation of clinical infections and associated prescribing proportions correctly. Time (in minutes) of travel from the patient’s home to the facility will be collected for each patient to understand if there are any prescribing differences based on how far the patient had to travel to the facility. Information will also be collected as to whether the patient had previously sought medical care / medication for this infection episode.

Any relevant comorbidities that may influence antibiotic prescribing practices or clinical severity of infections will be collected as below with additional free text provision to specify any not included in this list:

•   HIV

•   Malnutrition

•   Diabetes

•   Chronic obstructive pulmonary disease (COPD)

•   Asthma

•   Other chronic lung problems

•   Chronic heart problems

**Common infection symptoms and linked clinical diagnoses**

Key infections symptoms / diagnoses are based around clinical presentations described in the WHO EML Antibiotic Book. As this book provides model global guidance on prescribing for 35 common infections in adults and children, including the choice of drug, dose and duration, collecting infections and symptoms that can be mapped back to the Antibiotic Book and other guidelines is important to standardise data collection and allow for observed prescribing to be compared to guidelines.

For all primary presentations, additional symptoms are also identified including the presence of fever to assess severity of infection based around indications for antibiotic prescribing. The full list of primary infection symptoms / clinical diagnoses and additional symptom questions for each infection presentation can be found in Table 1.

**Antibiotics of interest**

We are interested in capturing all systemic antibiotics (e.g oral, intramuscular, intravenous) and topical antibiotics prescribed in primary healthcare for a given infection episode after consultation. All antivirals, antifungals, anthelminthics, antimalarials, medicines for HIV, and any antibiotics eye drops are excluded. Generic name of antibiotic, prescribed dose and prescribed duration will be collected.

Case report forms (CRFs) for consultation data are available in extended data online repository.

### 5.5.3 Pragmatic approach to data collection

Given the different structures of primary healthcare facilities participating in our study and the structures of the study teams, we allowed for pragmatic approaches to data collection to ensure the study was feasible in different settings. Data was collected electronically in ODK Collect (see below); however sites were given the option to collect data directly into ODK or to use paper CRFs if they preferred and then enter the data in ODK Collect at a later time. Sites were encouraged to enter data directly into ODK as much as possible to reduce the time burden on data collection teams. Sites were also given flexibility in who collected the data. Data could be collected directly by the prescriber/dispenser on paper CRFs or in ODK, or by having an observer record the consultation data during the consultation or between the consultations. Sites were given an option to collect data from the medical notes as a last option; however, this was only allowed where symptoms were routinely and comprehensively enough recorded in the medical notes to allow for retrieval. The study team at CSG worked with site teams before and during the study to find the best approach for each team. Data were collected on the day of each survey regarding the methods of data collection used which can be used in analysis and to assess feasibility of the methodology.

### 5.5.4 Data management, protection and security

Data will be collected on devices using the ODK Collect app. Only users at sites authorised by the central Project Team at CSG, who have been allocated a QR code for the study and will be able to use the application and collect data. All patient data collected will be completely anonymous and there will be no unique identifiable patient data captured for any participant. Codebooks for electronic data capture in ODK (XLS forms) are available in the APC-PPS online repository.

Data is encrypted in ODK Collect app and is sent encrypted to the ODK Cloud server. Data collected by sites will be stored centrally on ODK Cloud server based in an EU data centre, hosted by ODK. The server will be private to the central Project Team members based at CSG and University of Oxford, and no unauthorised users will have access to the central server. The server is GDPR compliant and ISO27K, CSA STAR and SOC 2 certified. The server is backed up continuously and each back up is stored for 30 days. Data are encrypted on the ODK Central server at rest.

The data will also be downloaded from ODK Cloud monthly to a secure server based at City St. George’s, University of London, and there will be encrypted back up tapes, with 24-hour security. CSG servers which are backed up overnight, every night, to hard disk and then cloned to tape storage. Full backups of all the data are carried out monthly. The cloned tapes are stored separately in a fireproof and bombproof safe off-site.

### 5.5.5 Data analysis

Data will be analysed descriptively in the first instance. Rates of presentation for each clinical infection will be calculated. Overall antibiotic prescribing proportion and overall proportions by AWaRe categories will be summarised. The antibiotic prescribing proportion and AWaRe categories will be summarised for each clinical infection and be compared with the new WHO AWaRe Antibiotic Book prescribing guidelines, peers, and ideal prescribing proportions. Factors including demographics (e.g. sex, age, time to health facility), comorbidities and infection severity will be explored to understand how they relate to an antibiotic prescription for an encounter, where sufficient data exists.

These data will also be used in simulations to explore the ideal sample size and sampling frame that would be needed for surveillance of antibiotic use in primary healthcare including the number and type of sites in a country, number of patients per site and frequency of sampling to inform future surveillance using PPS. We will aim to use these data to inform sample size calculations, understand co-efficients of variation and how to extrapolate from the number of patients per PPS time unit.

## 5.6 Ethical and regulatory considerations

### 5.6.1 Research Ethics Committee (REC) and other Regulatory review & reports

Before the start of the study, ethical approval was obtained for this study protocol from City St George’s University of London ethics committee (REC 2022.0170). Individual sites are responsible for ensuring relevant local and national ethics and regulatory approvals are in place prior to starting the study. The Project Team at CSG supported sites in preparation of documents required to meet these obligations. No site started the study without having the appropriate approvals in place.

### 5.6.2 Amendments

For any amendment to the study, the Chief Investigator or delegated personnel, in agreement with the sponsor will submit information to the appropriate body to issue approval for the amendment. The Chief Investigator or delegated personnel will work with sites so they can put the necessary arrangements in place to implement the amendment to confirm their support for the study as amended.

### 5.6.3 Protocol compliance

Protocol deviations, or data breaches are departures from the approved protocol. Any protocol deviations and data breaches must be adequately documented on the relevant forms and reported to the Chief Investigator and Sponsor in a timely manner. However, overall risk of deviations and data breaches are low given the study design and risk to participants is negligible as there is no identifiable or personal data collected.

### 5.6.4 Archiving Arrangements

The full dataset will be downloaded from ODK Cloud at the end of data collection and the final dataset will be deposited on the City St. George’s Research Data Repository upon study completion. Analysis code and reports will also be archived on the APC-PPS Project space on the City St. George’s Research Data Repository. Access to the raw, unaggregated data on the repository will be restricted to only the central Project Team members at CSG and University of Oxford. Sites will not be able to view/access raw data on the repository; but will be returned their raw data separately. Data will not be used for any purposes beyond the current described APC-PPS project and the ADILA project, without explicit permission and data sharing agreements in place with each individual site. Any aggregate datasets, code used for summary analyses and data visualisations / dashboards will be available to view on the repository Project space by collaborators at site teams 6–12 months after study completion after summary data has been returned to sites and the main paper(s) submitted for publication.

Datasets will be retained on the repository for the duration of the data retention period of 5 years. Any aggregated datasets that have been made publicly available will be published with a DOI and retained in perpetuity. All datasets archived on the repository will be accompanied by corresponding metadata in the study’s documentation and cataloguing standards.

The project coordinator as CSG will be responsible for uploading the final datasets, reports and code to the repository. The project coordinator will also be responsible for maintaining accuracy, completeness, relevance and timelines of all data archived on the repository. The City St George’s Research Data Service will support the project coordinator where required and ensure the data is preserved to the highest available standards. The Chief Investigator will have overall responsibility for the data archived in the repository. For corporate information governance purposes, the PI will be considered the data owner and will be the main contact for the data for the duration of the retention period. If the PI leaves City St George’s his/her institute manager will be responsible for the archived data.

## 5.7 Dissemination and publication policy

### 5.7.1 Guiding principles

The results of this study will be published in scientific and academic peer-reviewed journals and submitted as abstracts and presentations to relevant international conferences. The site and country collaborators will be involved in developing and reviewing drafts of manuscripts, abstracts and other publications and presentations arising from the results of this project.

The dissemination and publication policy for the APC-PPS project is guided by two overarching principles:

1. Transparency – All sites contributing data to the project will be informed as to the use of their data. We will ensure a process is in place for sites and collaborators to access data from the APC-PPS project for the purpose of generating local abstracts, reports, presentations and publications and the process of project approval for subsequent work are clear and agreed by all participating members of the project.
2. Quality – We will maintain a centralised publication and abstract discussion and approval process within the APC-PPS project to maintain a high quality of overall scientific output.

### 5.7.2 Site data at the end of study

Individual sites will receive access to a summary report of their data and their raw data within 6–12 months of the end of the project (defined as end of data collection activities for all sites). Per individual agreements with each site, summary data will be shared separated by (anonymised) site, country and overall which will be published on the APC-PPS Project space of the City St. George’s Research Data Repository at the end of the project and included in the analyses for the wider ADILA project. Access to raw individual site data by the country coordinator(s) will only be with explicit permission from each site in that country. Publications and other outputs from the wider ADILA project that use data contributed by collaborators from the APC-PPS will be governed by the ADILA project publication policy (separate attachment). Sites will have an opportunity to opt out of their data being used in the wider ADILA project for analyses beyond those described in the APC-PPS protocol.

### 5.7.3 Abstracts and papers authorship

In general, all authors publishing abstracts and papers using data from the APC-PPS project are expected to adhere to the ICMJE authorship guidelines which includes:

- Substantial contributions to the conception or design of the work; or the acquisition, analysis, or interpretation of data for the work; AND
- Drafting the work or revising it critically for important intellectual content; AND
- Final approval of the version to be published; AND
- Agreement to be accountable for all aspects of the work in ensuring that questions related to the accuracy or integrity of any part of the work are appropriately investigated and resolved.

The full dissemination and publication policy for APC-PPS is available in the extended data.

## Ethics and consent

Before the start of the study, ethical approval was obtained for this study protocol from City St George’s University of London ethics committee (REC 2022.0170, 07/11/2022). Individual sites are responsible for ensuring relevant local and national ethics and regulatory approvals are in place prior to starting the study. The Project Team at CSG supported sites in preparation of documents required to meet these obligations. No site started the study without having the required approvals in place.

Data collected for this study will be fully anonymised at the time of data capture and will not contain any personal or identifiable information and clinics are only identified by a facility ID code in the data. In many settings, these types of data would be routinely collected as part of a clinical audit to assess adherence to local guidelines so we will not be obtaining individual informed consent from the patients for the purposes of this study. This is also to ensure feasibility by reducing the disruption to clinics and routine practice as formal written consent is more time burdensome and disruptive to routine practice. To understand prescribing practices in primary care, it is essential to capture as many patients presenting as possible to understand the true rates of different infections and true prescribing rates for each type of infection; therefore reducing burden on sites and disruption to patient visits was essential to ensure representative sampling.

All ethics committees approved the protocol as described without requiring formal written consent.

## Data Availability

No data are associated with this article. The following supplementary materials are available in the St George’s University of London FigShare Research Data Repository. St George’s, University of London FigShare Research Data Repository: APC-PPS Protocol and Protocol Synopsis https://doi.org/10.24376/rd.sgul.27325758 ^17^. This project contains the following extended data: pps_synopsis_v2.0_28Oct22.pdf adila_apc_protocol_v2.0_28Oct22_signed.pdf This is the protocol and protocol synopsis for the Antibiotics in Primary Care Point Prevalence Survey (APC-PPS). St George’s, University of London FigShare Research Data Repository: Data collection forms for the APC-PPS. https://doi.org/10.24376/rd.sgul.27325332 ^18^. This project contains the following extended data: Consultation Level Data CRF v2.0 01.05.2023 2 pages.pdf Facility Information antibiotic availability v1.0 20.04.2023.xlsx Facility Information CRF v1.0 26.04.2023.pdf Survey Data CRF v1.0 11.04.2023.pdf These are the data collection forms (CRFs) for the Antibiotics in Primary Care Point Prevalence Survey (APC-PPS). There are three levels: 1. Facility form (to be completed once in the study period); 2. Survey level form (to be completed on every day the survey is conducted); 3. Consultation-level form (to be completed for every eligible consultation). Additionally, the Antibiotic Availability form (excel) is completed once in the study period. St George’s, University of London FigShare Research Data Repository: Data Codebook for ODK electronic data capture. https://doi.org/10.24376/rd.sgul.27325695 ^19^. This project contains the following extended data: ODK XLSForm APC PPS Consultation Level Data v1.3 30.08.2023.xlsx ODK XLSForm APC PPS Facility Information v1.0 12.04.2023.xlsx ODK XLSForm APC PPS Survey Data v1.0 12.04.2023.xlsx These are the XLS forms used for electronic data capture via ODK (Open Data Kit) for the Antibiotics in Primary Care Point Prevalence Survey (APC-PPS). There are codebooks for each of the three CRFs to be completed in the study: 1. Facility form (to be completed once in the study period); 2. Survey level form (to be completed on every day the survey is conducted); 3. Consultation-level form (to be completed for every eligible consultation). St George’s, University of London FigShare Research Data Repository: E-Learning Platform on The Global Health Network. https://doi.org/10.24376/rd.sgul.27325728 ^20^. This is the link to the e-learning platform of the Antibiotics in Primary Care Point Prevalence Survey (APC-PPS) e-Learning Platform on The Global Health Network (TGHN). The materials were designed by the APC-PPS Project Team at SGUL and the web platform and module design by the team at TGHN. St George’s, University of London FigShare Research Data Repository: Additional training materials for the APC-PPS. https://doi.org/10.24376/rd.sgul.27325707 ^21^. This project contains the following extended data: APC-PPS Training Manual - Eligibility v0.3 231110.pdf APC-PPS Training Manual - How to do web-based data entry 19.04.2023.pdf APC-PPS Training Manual - How to set up ODK Collect for data collection 17.02.2023.pdf APC-PPS Training Manual - How to submit a form in ODK 17.02.2023 (1).pdf APC-PPS Training Manual - Study Design v0.2 231110.pdf These are the training materials used for the Antibiotics in Primary Care Point Prevalence Survey (APC-PPS). They contain guidance on using ODK via the app and via the web, how to submit a form on ODK, and refresher guides on Eligibility and Study Design. St George’s, University of London FigShare Research Data Repository: Publication policy for APC-PPS. https://doi.org/10.24376/rd.sgul.27325725 ^22^. This project contains the following extended data: apc_pps_pubpolicy_v1.1_15May23 - Master.pdf The Master Publication Policy of the Antibiotics in Primary Care Point Prevalence Survey (APC-PPS). **Data are available under the terms of the C reative C ommons A ttribution 4.0 I nternational L icense (CC-BY 4.0).**
