# Supplementary material for: A pilot protocol for surveillance of infection and antibiotic prescribing in primary healthcare across the globe: Antibiotic Prescribing in Primary Healthcare Point Prevalence Survey (APC-PPS)
Source: Wellcome Open Res. 2026 Mar 31;10:26. Originally published 2025 Jan 21. [Version 2] doi: 10.12688/wellcomeopenres.23420.2 (PMC12397736; doi:10.12688/wellcomeopenres.23420.2)
Supplement: Peer review history

# Reviewer report: Reviewer response for version 2

**DOI:** 10.21956/wellcomeopenres.28872.r152255

**Published:** 2026-04-24

**Authors:** Lars Småbrekke

**Affiliations:** 1 Clinical Pharmacy and Pharmacoepidemiology, Department of Pharmacy, Faculty of Health Sciences, UiT Arctic University of Norway, Tromsø, Norway

The author response is clear and comprehensive, and I approve the revision of the manuscript.

 However, I have one additional comment regarding the patient level data collected. The author could consider including information on whether the visit is a revisit due to treatment failure, and if so, also collect data on the choice of initial treatment.

Is the study design appropriate for the research question?

Partly

Is the rationale for, and objectives of, the study clearly described?

Yes

Are sufficient details of the methods provided to allow replication by others?

Yes

Are the datasets clearly presented in a useable and accessible format?

Not applicable

Reviewer Expertise:

Pharmacoepidemiology; Clinical Pharmacy;

I confirm that I have read this submission and believe that I have an appropriate level of expertise to confirm that it is of an acceptable scientific standard.

# Reviewer report: Reviewer response for version 1

**DOI:** 10.21956/wellcomeopenres.25830.r127051

**Published:** 2025-08-29

**Authors:** Kevin L Schwartz

**Affiliations:** 1 University of Toronto, Toronto, Ontario, Canada

This is a protocol for a point prevalence survey of antibiotic prescribing in primary care clinics from LMICs. According to this protocol, which appears published online, the actual study was conducted and completed in November 2024.

 The study's primary objective is feasibility. The authors have not outlined the primary feasibility outcomes to be collected. Specifically, they have not defined the actual measures of feasibility, expected numbers or metrics that will measure/define as feasible.

 Otherwise I have no concerns with the protocol. Aside from being reviewed after study completion it is clearly written and an important study.

Is the study design appropriate for the research question?

Yes

Is the rationale for, and objectives of, the study clearly described?

Yes

Are sufficient details of the methods provided to allow replication by others?

Yes

Are the datasets clearly presented in a useable and accessible format?

Not applicable

Reviewer Expertise:

Infectious Diseases, Antimicrobial Stewardship, Epidemiology

I confirm that I have read this submission and believe that I have an appropriate level of expertise to confirm that it is of an acceptable scientific standard.

# Response

**Published:** 2026-03-10

**Authors:** Aislinn Cook

**Affiliations:** Centre for Neonatal and Paediatric Infection, St George's University of London, London, England, UK

Thank you for your comments. I have responded to them below.  This is a protocol for a point prevalence survey of antibiotic prescribing in primary care clinics from LMICs. According to this protocol, which appears published online, the actual study was conducted and completed in November 2024. The study was delayed in some facilities and data collection was not completed until partway through 2025. Analysis is currently underway.

 The study's primary objective is feasibility. The authors have not outlined the primary feasibility outcomes to be collected. Specifically, they have not defined the actual measures of feasibility, expected numbers or metrics that will measure/define as feasible. Thank you for this comment. This is helpful. As this is a pilot, feasibility will be assessed in several ways. One will be the broad participation in the study – how many facilities participate and complete all 8 surveys in different settings. From a data collection perspective we will use the number of eligible patients compared to the number of patients for which data was collected as metric of data collection feasibility. We will also use the method and person doing data collection which was collected as part of each survey to assess personnel required and ability to collect these data during consultations. We will also use the time taken to do data entry for each record from the metadata from ODK. We will send a feasibility survey at the end of the study to better capture self-reported measures of how easy it was to find data, do data entry, whether there was any disruption to clinics. During the study, we had regular contact with participating sites and recorded meeting notes of challenges discussed and solutions found. We allowed for unknown to be selected for all fields and we will use percentage of missingness for key fields as an assessment of data availability triangulated with method of data collection.

# Response

**Published:** 2026-03-30

**Authors:** Aislinn Cook

**Affiliations:** Centre for Neonatal and Paediatric Infection, St George's University of London, London, England, UK

Thank you for you comments. I have responded below in bold

**This is a protocol for a point prevalence survey of antibiotic prescribing in primary care clinics from LMICs. According to this protocol, which appears published online, the actual study was conducted and completed in November 2024.**

 The study was delayed in some facilities and data collection was not completed until partway through 2025. Analysis is currently underway.

**The study's primary objective is feasibility. The authors have not outlined the primary feasibility outcomes to be collected. Specifically, they have not defined the actual measures of feasibility, expected numbers or metrics that will measure/define as feasible.**

 Thank you for this comment. This is helpful. As this is a pilot, feasibility will be assessed in several ways. One will be the broad participation in the study – how many facilities participate and complete all 8 surveys in different settings. From a data collection perspective we will use the number of eligible patients compared to the number of patients for which data was collected as metric of data collection feasibility. We will also use the method and person doing data collection which was collected as part of each survey to assess personnel required and ability to collect these data during consultations. We will also use the time taken to do data entry for each record from the metadata from ODK. We will send a feasibility survey at the end of the study to better capture self-reported measures of how easy it was to find data, do data entry, whether there was any disruption to clinics. During the study, we had regular contact with participating sites and recorded meeting notes of challenges discussed and solutions found. We allowed for unknown to be selected for all fields and we will use percentage of missingness for key fields as an assessment of data availability triangulated with method of data collection.

# Reviewer report: Reviewer response for version 1

**DOI:** 10.21956/wellcomeopenres.25830.r127054

**Published:** 2025-08-12

**Authors:** Margaret Lubwama

**Affiliations:** 1 Medical Microbiology, Makerere University College of Health Sciences (Ringgold ID: 58589), Kampala, Central Region, Uganda

Thank you for the invitation to review this study protocol.

 This is an important study in this era of antimicrobial resistance (AMR). Given that the highest burden of AMR is in low- and middle-income countries (LMICs), and antibiotic overuse and misuse are among the drivers of AMR, it is of utmost importance that we understand the antibiotic prescribing practices and antibiotic use in primary healthcare in LMICs. 

**General comments**

 How does the WHO AWaRe guidelines affect the national clinical guidelines of the different countries? Are there any similarities or differences that may affect the prescribing practices of the different health facilities in the different countries? How well do the national clinical guidelines match the WHO AWaRe guidelines. This can guide in policy decisions. How is this study taking this into account?

**Section 5.4**

 Is this study a cross-sectional study collecting data at different points in time. Can the authors clarify this?

 What proportions of private and public facilities will be included for the different countries? Will it be representative of the facilities in the different countries?

 The team has clearly outlined infection symptoms/diagnoses of interest which will be used for the eligibility criteria. It might be that the list provided by the study collects much more data than what the healthcare centers are used to collecting. This may require more time. How many patients are estimated per healthcare center? Will all the data on the list be collected in 4 hours? While the authors have mentioned that it may not be possible to know the sample size per clinic, including the number of patients estimated per healthcare center, and the amount of time it will take to complete screening for each patient will provide insight into the feasibility of 4 hours. Is it possible to get these estimates from the records in the different healthcare facilities?

 While the authors mention that they will not be obtaining individual informed consent from the patients for the purposes of this study, they may need a waiver of consent from the respective IRBs. Was this obtained if the IRBs require it?

**Section 5.5.4**

 How will the data collected in the CRFs be managed? Where will the CRFs be stored? Who will be responsible for the CRFs?

 If phones go missing what is the risk of data leaking. What precautions have been taken to address this? This can be included in a risk analysis plan.

**Section 5.5.5**

 How will infection severity be determined and measured in this study?

 I agree with the authors that it is important to understand how the different factors are associated with antibiotic prescribing practices. However, it is not very clear which statistical method will be used to address this. A more detailed statistical analysis plan is needed to show how the associations will be measured in this study.

Is the study design appropriate for the research question?

Yes

Is the rationale for, and objectives of, the study clearly described?

Yes

Are sufficient details of the methods provided to allow replication by others?

Partly

Are the datasets clearly presented in a useable and accessible format?

Not applicable

Reviewer Expertise:

Medical microbiology, antimicrobial resistance (antibacterial)

I confirm that I have read this submission and believe that I have an appropriate level of expertise to confirm that it is of an acceptable scientific standard, however I have significant reservations, as outlined above.

# Response

**Published:** 2026-03-10

**Authors:** Aislinn Cook

**Affiliations:** Centre for Neonatal and Paediatric Infection, St George's University of London, London, England, UK

Thank you for your thorough review and comments. I have answered your comments below. 

 How does the WHO AWaRe guidelines affect the national clinical guidelines of the different countries? Are there any similarities or differences that may affect the prescribing practices of the different health facilities in the different countries? How well do the national clinical guidelines match the WHO AWaRe guidelines. This can guide in policy decisions. How is this study taking this into account?

 Thank you for this important question. The WHO AWaRe book is an official WHO guideline which can fill gaps where national treatment guidelines aren’t available or up to date in countries. Countries and clinics that have their own guidelines will be expected to be following those as part of routine practice. The AWaRe Book provides a global framework to compare to across diverse settings, however, we are aware that any comparison to global guidelines may not account for differences in local treatment guidelines. A recent analysis did find quite high agreement in first line antibiotic choices in the AWaRe Book and local treatment guidelines for paediatric infections (Donà D, et al. eClinicalMedicine. DOI: 10.1016/j.eclinm.2025.103437) across 80 countries for primary care infections. We acknowledge that expected antibiotic prescribing patterns may be different than we estimate using AWaRe Book guidance compared to national treatment guidelines if they differ but given the broad number of countries and facilities included, we won’t be able to adapt this for the global analysis. However, we will look to provide guidance to our country partners for their analysis to compare prescribing patterns to their own treatment guidelines using the same framework as will be used for the AWaRe Book comparison.   Is this study a cross-sectional study collecting data at different points in time. Can the authors clarify this? This study is a cross-sectional study collecting data at half-day intervals for 8 repeated time points in sets of two surveys as shown in Figure 1.

 What proportions of private and public facilities will be included for the different countries? Will it be representative of the facilities in the different countries? This is a convenience sample as this is a pilot study and the participation of public and private facilities will depend on the country involved. Generally, it is more difficult to engage private facilities as there are less incentives to provide these types of data and concerns that it will be used by authorities for enforcement of practices. We tried to engage both types of facilities but expect it will be more public facilities that participate in the study. As this is a pilot of the methodology, it will likely not be representative of the facilities and broad care seeking behaviours in the different countries. The level to which it is or is not representative will depend on the structure of the primary healthcare system in each country and healthcare seeking behaviours.  

   The team has clearly outlined infection symptoms/diagnoses of interest which will be used for the eligibility criteria. It might be that the list provided by the study collects much more data than what the healthcare centers are used to collecting. This may require more time. How many patients are estimated per healthcare center? Will all the data on the list be collected in 4 hours? While the authors have mentioned that it may not be possible to know the sample size per clinic, including the number of patients estimated per healthcare center, and the amount of time it will take to complete screening for each patient will provide insight into the feasibility of 4 hours. Is it possible to get these estimates from the records in the different healthcare facilities?

 Thank you for your important questions. As this was a pilot study of a novel methodology, we did not limit participation to clinics based on the size of the clinic or the number of patients estimated to visit. We collected this information as part of the pilot study – total patient visiting, total with infections and the number included – to enable exploration of minimum sample sizes needed for future studies. The data collected was data routinely needed during a consultation and the expectation was that this would be available during the study data collection period. For fields that were not available, data collectors were able to use medical notes/records where possible to fill in data such as age, etc or record unknown for fields that were not available. By including a diverse range of primary care facilities, the structure and flow of patients through clinics was different so we work closely with clinics to overcome any challenges with data collection and to minimise disruption to routine care. In some instances, not all data was available in the four hour period – i.e. if patients were triaged with symptoms collected, then sent for labs and returned later to have a prescribing decision made – in these instances, data on antibiotic prescribing was captured after the four hour period but still related to only consultations from the initial four hour period. We did not require any data collection to happen beyond the single day of the survey (e.g. if patients returned the next day for a prescribing decision). How will the data collected in the CRFs be managed? Where will the CRFs be stored? Who will be responsible for the CRFs?

 Thank you for your comment. The data collection procedures are outlined in section 6.5.3 and 6.5.4. The data in the electronic CRF was managed by a data manager at City St George’s of London using ODK Cloud. Data was backed up regularly by ODK Cloud and downloaded monthly to a secure server at City St George’s. If data collectors used paper CRFs to collect data during the consultation, the site PI or country lead (depending on the structure of the team) was responsible for the storage of these CRFs and their destruction at the end of the study. No identifiable information was collected and all data was collected anonymously therefore it was not possible to link any paper CRFs directly to entries in ODK after data entry. Countries had separate data entry platforms to keep their data separate and each user had a unique QR code. If phones go missing what is the risk of data leaking. What precautions have been taken to address this? This can be included in a risk analysis plan. Country partners used dedicated study tablets or phones. Tablets and devices were password protected with the code known only to the data collector. All data was anonymously collected and clinics themselves were identified using a random identifier with the key kept separately to the clinical data collected. It was not possible for study teams to identify individuals in the data once the data had been sent to ODK and only the data collectors and the central study team knew which facility identifier related to which facility further reducing the risk if phones were to be missing or logged into.   How will infection severity be determined and measured in this study?

 Infection severity will be determined using a combination of fields such as presence of fever, presence of the follow up symptoms, age and comorbidities. The follow up symptoms such as presence of shortness of breath, chest pain, long cough for patients presenting with acute cough will be used to guide severity. It is of course not possible to fully determine severity beyond the fields available and that we don’t have direct assessment of the clinicians who saw each patient. We will use presence of comorbidities, age, sex and pregnancy as well to determine severity and necessity of antibiotic prescription using the WHO AWaRe Book risk guidance.     However, it is not very clear which statistical method will be used to address this. A more detailed statistical analysis plan is needed to show how the associations will be measured in this study.

 Thank you for this comment. At the time of protocol development given this is a pilot study, we did not know whether there would be sufficient data to assess these factors and sufficient data from within countries and similar types of clinics to assess factors. We will be looking at guideline compliance with the WHO AWaRe Book in the first instance and incorporate data on antibiotic availability and pricing to compare observed vs. expected prescribing patterns within each clinic. Observed vs expected prescribing proportions, AWaRe proportions and specific antibiotic proportions will be summarised as proportions with binomial confidence intervals. Beyond that, where there is sufficient variability in case-mix and number of clinics in a country, we will explore the influence of case-mix on differences in prescribing choices between facilities within a country using causal variance decomposition. Additionally, where data allows, we will use a within-between model to look at patient and facility level factors that explain prescribing propensity.

# Response

**Published:** 2026-03-30

**Authors:** Aislinn Cook

**Affiliations:** Centre for Neonatal and Paediatric Infection, St George's University of London, London, England, UK

Thank you for your comments and review. I have responded below in black to your comments (in bold).   

**General comments**

**How does the WHO AWaRe guidelines affect the national clinical guidelines of the different countries? Are there any similarities or differences that may affect the prescribing practices of the different health facilities in the different countries? How well do the national clinical guidelines match the WHO AWaRe guidelines. This can guide in policy decisions. How is this study taking this into account?**

 Thank you for this important question. The WHO AWaRe book is an official WHO guideline which can fill gaps where national treatment guidelines aren’t available or up to date in countries. Countries and clinics that have their own guidelines will be expected to be following those as part of routine practice. The AWaRe Book provides a global framework to compare to across diverse settings, however, we are aware that any comparison to global guidelines may not account for differences in local treatment guidelines. A recent analysis did find quite high agreement in first line antibiotic choices in the AWaRe Book and local treatment guidelines for paediatric infections (Donà D, et al. eClinicalMedicine. DOI: 10.1016/j.eclinm.2025.103437) across 80 countries for primary care infections. We acknowledge that expected antibiotic prescribing patterns may be different than we estimate using AWaRe Book guidance compared to national treatment guidelines if they differ but given the broad number of countries and facilities included, we won’t be able to adapt this for the global analysis. However, we will look to provide guidance to our country partners for their analysis to compare prescribing patterns to their own treatment guidelines using the same framework as will be used for the AWaRe Book comparison.

**Section 5.4**

**Is this study a cross-sectional study collecting data at different points in time. Can the authors clarify this? **This study is a cross-sectional study collecting data at half-day intervals for 8 repeated time points in sets of two surveys as shown in Figure 1.

**What proportions of private and public facilities will be included for the different countries? Will it be representative of the facilities in the different countries? **This is a convenience sample as this is a pilot study and the participation of public and private facilities will depend on the country involved. Generally, it is more difficult to engage private facilities as there are less incentives to provide these types of data and concerns that it will be used by authorities for enforcement of practices. We tried to engage both types of facilities but expect it will be more public facilities that participate in the study. As this is a pilot of the methodology, it will likely not be representative of the facilities and broad care seeking behaviours in the different countries. The level to which it is or is not representative will depend on the structure of the primary healthcare system in each country and healthcare seeking behaviours.  

**The team has clearly outlined infection symptoms/diagnoses of interest which will be used for the eligibility criteria. It might be that the list provided by the study collects much more data than what the healthcare centers are used to collecting. This may require more time. How many patients are estimated per healthcare center? Will all the data on the list be collected in 4 hours? While the authors have mentioned that it may not be possible to know the sample size per clinic, including the number of patients estimated per healthcare center, and the amount of time it will take to complete screening for each patient will provide insight into the feasibility of 4 hours. Is it possible to get these estimates from the records in the different healthcare facilities?** Thank you for your important questions. As this was a pilot study of a novel methodology, we did not limit participation to clinics based on the size of the clinic or the number of patients estimated to visit. We collected this information as part of the pilot study – total patient visiting, total with infections and the number included – to enable exploration of minimum sample sizes needed for future studies. The data collected was data routinely needed during a consultation and the expectation was that this would be available during the study data collection period. For fields that were not available, data collectors were able to use medical notes/records where possible to fill in data such as age, etc or record unknown for fields that were not available. By including a diverse range of primary care facilities, the structure and flow of patients through clinics was different so we work closely with clinics to overcome any challenges with data collection and to minimise disruption to routine care. In some instances, not all data was available in the four hour period – i.e. if patients were triaged with symptoms collected, then sent for labs and returned later to have a prescribing decision made – in these instances, data on antibiotic prescribing was captured after the four hour period but still related to only consultations from the initial four hour period. We did not require any data collection to happen beyond the single day of the survey (e.g. if patients returned the next day for a prescribing decision).

**Section 5.5.4**

**How will the data collected in the CRFs be managed? Where will the CRFs be stored? Who will be responsible for the CRFs?** Thank you for your comment. The data collection procedures are outlined in section 6.5.3 and 6.5.4. The data in the electronic CRF was managed by a data manager at City St George’s of London using ODK Cloud. Data was backed up regularly by ODK Cloud and downloaded monthly to a secure server at City St George’s. If data collectors used paper CRFs to collect data during the consultation, the site PI or country lead (depending on the structure of the team) was responsible for the storage of these CRFs and their destruction at the end of the study. No identifiable information was collected and all data was collected anonymously therefore it was not possible to link any paper CRFs directly to entries in ODK after data entry. Countries had separate data entry platforms to keep their data separate and each user had a unique QR code.

**If phones go missing what is the risk of data leaking. What precautions have been taken to address this? This can be included in a risk analysis plan.** Country partners used dedicated study tablets or phones. Tablets and devices were password protected with the code known only to the data collector. All data was anonymously collected, and clinics themselves were identified using a random identifier with the key kept separately to the clinical data collected. It was not possible for study teams to identify individuals in the data once the data had been sent to ODK and only the data collectors and the central study team knew which facility identifier related to which facility further reducing the risk if phones were to be missing or logged into.

**Section 5.5.5**

**How will infection severity be determined and measured in this study?**

 Infection severity will be determined using a combination of fields such as presence of fever, presence of the follow up symptoms, age and comorbidities. The follow up symptoms such as presence of shortness of breath, chest pain, long cough for patients presenting with acute cough will be used to guide severity. It is of course not possible to fully determine severity beyond the fields available and that we don’t have direct assessment of the clinicians who saw each patient. We will use presence of comorbidities, age, sex and pregnancy as well to determine severity and necessity of antibiotic prescription using the WHO AWaRe Book risk guidance.

**I agree with the authors that it is important to understand how the different factors are associated with antibiotic prescribing practices. However, it is not very clear which statistical method will be used to address this. A more detailed statistical analysis plan is needed to show how the associations will be measured in this study.** Thank you for this comment. At the time of protocol development given this is a pilot study, we did not know whether there would be sufficient data to assess these factors and sufficient data from within countries and similar types of clinics to assess factors. We will be looking at guideline compliance with the WHO AWaRe Book in the first instance and incorporate data on antibiotic availability and pricing to compare observed vs. expected prescribing patterns within each clinic. Observed vs expected prescribing proportions, AWaRe proportions and specific antibiotic proportions will be summarised as proportions with binomial confidence intervals. Beyond that, where there is sufficient variability in case-mix and number of clinics in a country, we will explore the influence of case-mix on differences in prescribing choices between facilities within a country using causal variance decomposition. Additionally, where data allows, we will use a within-between model to look at patient and facility level factors that explain prescribing propensity.

# Reviewer report: Reviewer response for version 1

**DOI:** 10.21956/wellcomeopenres.25830.r117674

**Published:** 2025-02-12

**Authors:** Lars Småbrekke, Robert Nuifondieng Foncha

**Affiliations:** 1 Clinical Pharmacy and Pharmacoepidemiology, Department of Pharmacy, Faculty of Health Sciences, UiT Arctic University of Norway, Tromsø, Norway; 2 Department of Pharmacy, UiT The Arctic University of Norway Faculty of Health Sciences (Ringgold ID: 60482), Tromsø, Troms, Norway

Thank you for the invitation to review this paper on surveillance of infections and antibiotic prescribing in primary health care in LMIC countries in Africa and Asia. This project will significantly contribute to our understanding of infection presentation and antibiotic prescribing across diverse settings.

**General Comments:**

 The sections on ethics, data collection platform, personnel training, data management, and consultation data are well-described. The volume of data collected is impressive.

 While we agree that point prevalence surveys (PPS) based on a convenience sample can provide insights into local diagnostic quality, therapy choices, and antibiotic prescriptions, it is unclear whether these results would be representative beyond the local or regional level. Additionally, there is a risk that participants may alter their behavior when they know they are being observed (Hawthorne effect). What measures are in place to mitigate these challenges?

**Chronological Comments:**

 In sections 5.1 and 5.2, you aim to quantify the frequency of different infection presentations and the proportion of patients receiving antibiotic prescriptions. We agree that this is a reasonable approach. However, in section 5.3, you state that the goal is to understand the relative presentation rate of clinical infections and antibiotic prescribing rates for these infections.

 Typically, a "rate" describes the occurrence of an event within a specific population over a defined period. It is usually expressed as a ratio where the numerator is the number of events (e.g., cases of a disease or patient encounters) and the denominator is the population at risk during that period. From the protocol, it is unclear how you define the population at risk and the risk period based on these point prevalence data. The concept of "rate" is repeated in sections 5.4.3.2 and 5.4.4.

 The sampling strategy aims to capture half a day of a standard clinic session using the ODK. Given the extensive data collection per patient encounter, it seems reasonable to restrict data collection to a four-hour period. However, it is unclear what the expected average time for completing data collection is and how this will be measured if data is not electronically collected during the consultation. Conducting all data collection during working hours will impact both employees and patients, potentially reducing the number of daily consultations. This will incur costs for the clinic and the patients (the "cost" for those who will not receive a consultation that day). How will these financial and ethical challenges be addressed?

 In section 5.5.2.3 (Consultation data collected), you state that age will be recorded in months for those ≤2 years and in years for those older. However, in Table 2 on PPS-specific information, you differentiate between adults (≥18 years) and children (<18 years). We commend the high resolution on age in the data and believe this should be explored further by investigating age groups <2 years, 2-5 years, 6-15 years, and >15 years(1). The periodic prevalence of treatment for young children is high in some areas, and aggregating into one group <18 years will result in a significant loss of information. In the list of relevant comorbidities, we suggest including TB.

 Consider recoding all generic names to ATC codes. This will facilitate analysis, allowing comparisons between regions with different prescribing patterns. It will also enable a comparison between the Defined Daily Dose (DDD) and the prescribed dose.

 In section 5.5.5 (Data analysis), it is clearly stated in the first sentence that the data will be analyzed descriptively. However, you also mention that with sufficient data, you will explore factors related to antibiotic prescriptions for an encounter, which will necessarily involve analytical statistics. We believe the protocol would benefit from a more detailed statistical analysis plan.

 A substantial amount of data is collected, and in our opinion, this resource will be underutilized if relying solely on descriptive measures. It is unclear whether representatives from the participating clinics are invited to participate in the project's evaluation.

Is the study design appropriate for the research question?

Partly

Is the rationale for, and objectives of, the study clearly described?

Yes

Are sufficient details of the methods provided to allow replication by others?

Yes

Are the datasets clearly presented in a useable and accessible format?

Not applicable

Reviewer Expertise:

Pharmacoepidemiology; Clinical Pharmacy;

We confirm that we have read this submission and believe that we have an appropriate level of expertise to confirm that it is of an acceptable scientific standard, however we have significant reservations, as outlined above.

## References

[1] : Age- and gender-specific antibacterial prescribing in Norway. *J Antimicrob Chemother* .2007;59(5) : 10.1093/jac/dkm032 971-6 10.1093/jac/dkm032 17329270

# Response

**Published:** 2026-03-10

**Authors:** Aislinn Cook

**Affiliations:** Centre for Neonatal and Paediatric Infection, St George's University of London, London, England, UK

Thank you for your comprehensive comments. I have split up your comment and my responses are below.   

 The sections on ethics, data collection platform, personnel training, data management, and consultation data are well-described. The volume of data collected is impressive.

 While we agree that point prevalence surveys (PPS) based on a convenience sample can provide insights into local diagnostic quality, therapy choices, and antibiotic prescriptions, it is unclear whether these results would be representative beyond the local or regional level. Thank you for your comment. The main findings from the study that will be applicable and representative beyond the local and regional level is the feasibility of the data collection tool. By working with partners in numerous clinics and at different levels of primary care (clinics, outpatients, community pharmacies) we will be able to provide a tool that works to collect basic clinical and prescribing data in diverse settings. By including a range of facilities and several facilities per country we think the study will demonstrate the applicability of this methodology to different settings. The findings will allow to us to compare similarities between countries and facility types within a country and to use a tool such as this for identifying conditions where interventions may provide the most improvement in inappropriate prescribing. Additionally, there is a risk that participants may alter their behavior when they know they are being observed (Hawthorne effect). What measures are in place to mitigate these challenges?

 Thank you for your comments, we agree that this can be cause for concern. We collect survey-level data on how the data were collected at each survey – from the notes, during consultation and by whom (observer, prescriber) for each survey so we can examine descriptively whether we see differences in the same facility when using different methods across different surveys and include this as a covariate in any modelling. We also did not collect any data on the prescriber themselves and ensured that this was clearly communicated to prescribers at all clinics where observation or prescriber-led data collection was conducted. However, given that many medical notes are not routinely completed in these settings or available after the consultation, direct observation/data collection during consultation is one of the few ways to get comprehensive data so it is not entirely possible to mitigate this bias.

 From the protocol, it is unclear how you define the population at risk and the risk period based on these point prevalence data. The concept of "rate" is repeated in sections 5.4.3.2 and 5.4.4.

 Thank you for this important comment. You are correct that we are focused on frequency rather than true rates. While we collect start and end time of the consultation period and the total number of patients that consult for any reason and total that consult for infection presentations, we do not have the full underlying population. We have updated the word rate to proportion to align with the data we have. However, it is unclear what the expected average time for completing data collection is and how this will be measured if data is not electronically collected during the consultation. We expected the average data collection to take 2-5 minutes depending on the flow of the consultation and when the data were collected within the consultation. Teams were given options for who and how the data could be collected, to better understand what was feasible and to enable broadest participation. Methodology for data capture was recorded for each survey. The data collectors, if not the prescriber, was able to capture data during the consultation itself, or from the medical notes where available (i.e. for age, etc). We asked partners at the end of the study the average time it took to complete the data collection when not recorded electronically and we are able to capture the time it took to complete data entry through metadata in ODK.

 Conducting all data collection during working hours will impact both employees and patients, potentially reducing the number of daily consultations. This will incur costs for the clinic and the patients (the "cost" for those who will not receive a consultation that day). How will these financial and ethical challenges be addressed?

 We worked closely with country partners to address any concerns about length of data collection to find methods that were the least disruptive. Partners were able to collect data in several ways depending on how the flow of the clinic worked, given the myriad ways primary healthcare clinics are structured in different countries and different levels of primary care. Partners could choose to collect data using observers so as to minimise disruption to the consultation (by not having the prescriber collect the data). Patients provided consent for an observer to be in the consultation. Where that was not possible, data was able to be collected from clinical notes between and after consultations provided there was enough clinical information recorded. We allowed for unknown to be entered for majority of fields to facilitate ease of data collection and to understand which fields are not routinely available in these settings. Where partners expressed concerns about impact on consultations during the study, we worked with them to find different solutions to data collection to prioritise minimising disruption to routine consultations.    

 In section 5.5.2.3 (Consultation data collected), you state that age will be recorded in months for those ≤2 years and in years for those older. However, in Table 2 on PPS-specific information, you differentiate between adults (≥18 years) and children (<18 years). We commend the high resolution on age in the data and believe this should be explored further by investigating age groups <2 years, 2-5 years, 6-15 years, and >15 years(1). The periodic prevalence of treatment for young children is high in some areas, and aggregating into one group <18 years will result in a significant loss of information. In the list of relevant comorbidities, we suggest including TB.

 Consider recoding all generic names to ATC codes. This will facilitate analysis, allowing comparisons between regions with different prescribing patterns. It will also enable a comparison between the Defined Daily Dose (DDD) and the prescribed dose.

 Thank you for this comment. We agree and we have collected data at the granular level (in months for children <2 years) and in years for everyone ≥2 years. We will explore age as a continuous variable in the first instance and consider grouping if needed based on granularity. Thank you for the suggestion regarding TB. We have included a space for “Other underlying conditions” for data collection and will consider explicitly adding TB in future versions. We will be mapping all generic names to ATC codes and AWaRe categories for comparison across groups and describing prescribed dosages.   We believe the protocol would benefit from a more detailed statistical analysis plan. A substantial amount of data is collected, and in our opinion, this resource will be underutilized if relying solely on descriptive measures. Thank you for your comments. As this was a pilot of a new methodology in different types of clinics with a set time period, it was not clear how many encounters we would get in similar types of settings and similar conditions. In order to compare prescribing practices between facilities, we will also need to ensure there is enough variability in patient case-mix in enough similar facility types in the same country which could not be defined prior to developing the protocol. If there is sufficient data for this analysis, we will analyse the clinic and patient-level factors related to the choice to prescribe and the choice of antibiotic, or AWaRe category using mixed-effect regression analysis. As outlined in the protocol we will also be evaluating guideline compliance with the WHO AWaRe Book guidance to compare observed prescribing patterns (the decision to prescribe and the choice of antibiotic, dose, duration when an antibiotic is prescribed). Data collected at the facility level on antibiotic availability and price will be incorporated into this analysis. Provided there is suitable variation in case-mix between facilities, further analyses comparing prescribing practices between clinics will be undertaken using causal variance decomposition. A more detailed statistical analysis plan will be developed prior to starting analysis. As part of a future master protocol building on this work, we will include this analysis plan and guidance defining minimum sample sizes.   It is unclear whether representatives from the participating clinics are invited to participate in the project's evaluation. Representatives from the countries and clinics participating in the research are invited to be part of the analysis and to use their own data for their own research questions. The results from each clinic will be fed back to country leads and clinic leads in the form of PDF reports of their common presentations, antibiotic prescribing practices as well as in the form of an interactive analytical dashboard that allows partners to explore their own datasets and use it for publications, reports and more to inform local research and policy interventions. We will also conduct a feasibility survey at the end of the study with participating data collectors at clinics and country leads to gain feedback on the challenges of implementation of this study.

# Response

**Published:** 2026-03-30

**Authors:** Aislinn Cook

**Affiliations:** Centre for Neonatal and Paediatric Infection, St George's University of London, London, England, UK

Thank you for your comments. For ease I have responded below to your comments (in bold) with my responses in black.   

**The sections on ethics, data collection platform, personnel training, data management, and consultation data are well-described. The volume of data collected is impressive.**

**While we agree that point prevalence surveys (PPS) based on a convenience sample can provide insights into local diagnostic quality, therapy choices, and antibiotic prescriptions, it is unclear whether these results would be representative beyond the local or regional level.** Thank you for your comment. The main findings from the study that will be applicable and representative beyond the local and regional level is the feasibility of the data collection tool. By working with partners in numerous clinics and at different levels of primary care (clinics, outpatients, community pharmacies) we will be able to provide a tool that works to collect basic clinical and prescribing data in diverse settings. By including a range of facilities and several facilities per country we think the study will demonstrate the applicability of this methodology to different settings. The findings will allow to us to compare similarities between countries and facility types within a country and to use a tool such as this for identifying conditions where interventions may provide the most improvement in inappropriate prescribing. **Additionally, there is a risk that participants may alter their behavior when they know they are being observed (Hawthorne effect). What measures are in place to mitigate these challenges?** Thank you for your comments, we agree that this can be cause for concern. We collect survey-level data on how the data were collected at each survey – from the notes, during consultation and by whom (observer, prescriber) for each survey so we can examine descriptively whether we see differences in the same facility when using different methods across different surveys and include this as a covariate in any modelling. We also did not collect any data on the prescriber themselves and ensured that this was clearly communicated to prescribers at all clinics where observation or prescriber-led data collection was conducted. However, given that many medical notes are not routinely completed in these settings or available after the consultation, direct observation/data collection during consultation is one of the few ways to get comprehensive data so it is not entirely possible to mitigate this bias.

**Chronological Comments:**

**In sections 5.1 and 5.2, you aim to quantify the frequency of different infection presentations and the proportion of patients receiving antibiotic prescriptions. We agree that this is a reasonable approach. However, in section 5.3, you state that the goal is to understand the relative presentation rate of clinical infections and antibiotic prescribing rates for these infections.**

**Typically, a "rate" describes the occurrence of an event within a specific population over a defined period. It is usually expressed as a ratio where the numerator is the number of events (e.g., cases of a disease or patient encounters) and the denominator is the population at risk during that period. From the protocol, it is unclear how you define the population at risk and the risk period based on these point prevalence data. The concept of "rate" is repeated in sections 5.4.3.2 and 5.4.4.**

 Thank you for this important comment. You are correct that we are focused on frequency rather than true rates. While we collect start and end time of the consultation period and the total number of patients that consult for any reason and total that consult for infection presentations, we do not have the full underlying population. We have updated the word rate to proportion to align with the data we have.

**The sampling strategy aims to capture half a day of a standard clinic session using the ODK. Given the extensive data collection per patient encounter, it seems reasonable to restrict data collection to a four-hour period. However, it is unclear what the expected average time for completing data collection is and how this will be measured if data is not electronically collected during the consultation.** We expected the average data collection to take 2-5 minutes depending on the flow of the consultation and when the data were collected within the consultation. Teams were given options for who and how the data could be collected, to better understand what was feasible and to enable broadest participation. Methodology for data capture was recorded for each survey. The data collectors, if not the prescriber, was able to capture data during the consultation itself, or from the medical notes where available (i.e. for age, etc). We asked partners at the end of the study the average time it took to complete the data collection when not recorded electronically and we are able to capture the time it took to complete data entry through metadata in ODK. **Conducting all data collection during working hours will impact both employees and patients, potentially reducing the number of daily consultations. This will incur costs for the clinic and the patients (the "cost" for those who will not receive a consultation that day). How will these financial and ethical challenges be addressed?**

 We worked closely with country partners to address any concerns about length of data collection to find methods that were the least disruptive. Partners were able to collect data in several ways depending on how the flow of the clinic worked, given the myriad ways primary healthcare clinics are structured in different countries and different levels of primary care. Partners could choose to collect data using observers so as to minimise disruption to the consultation (by not having the prescriber collect the data). Patients provided consent for an observer to be in the consultation. Where that was not possible, data was able to be collected from clinical notes between and after consultations provided there was enough clinical information recorded. We allowed for unknown to be entered for majority of fields to facilitate ease of data collection and to understand which fields are not routinely available in these settings. Where partners expressed concerns about impact on consultations during the study, we worked with them to find different solutions to data collection to prioritise minimising disruption to routine consultations.  

**In section 5.5.2.3 (Consultation data collected), you state that age will be recorded in months for those ≤2 years and in years for those older. However, in Table 2 on PPS-specific information, you differentiate between adults (≥18 years) and children (<18 years). We commend the high resolution on age in the data and believe this should be explored further by investigating age groups <2 years, 2-5 years, 6-15 years, and >15 years(1). The periodic prevalence of treatment for young children is high in some areas, and aggregating into one group <18 years will result in a significant loss of information. In the list of relevant comorbidities, we suggest including TB.**

**Consider recoding all generic names to ATC codes. This will facilitate analysis, allowing comparisons between regions with different prescribing patterns. It will also enable a comparison between the Defined Daily Dose (DDD) and the prescribed dose. **Thank you for this comment. We agree and we have collected data at the granular level (in months for children <2 years) and in years for everyone ≥2 years. We will explore age as a continuous variable in the first instance and consider grouping if needed based on granularity. Thank you for the suggestion regarding TB. We have included a space for “Other underlying conditions” for data collection and will consider explicitly adding TB in future versions. We will be mapping all generic names to ATC codes and AWaRe categories for comparison across groups and describing prescribed dosages.

**In section 5.5.5 (Data analysis), it is clearly stated in the first sentence that the data will be analyzed descriptively. However, you also mention that with sufficient data, you will explore factors related to antibiotic prescriptions for an encounter, which will necessarily involve analytical statistics. We believe the protocol would benefit from a more detailed statistical analysis plan.** A substantial amount of data is collected, and in our opinion, this resource will be underutilized if relying solely on descriptive measures. Thank you for your comments. As this was a pilot of a new methodology in different types of clinics with a set time period, it was not clear how many encounters we would get in similar types of settings and similar conditions. In order to compare prescribing practices between facilities, we will also need to ensure there is enough variability in patient case-mix in enough similar facility types in the same country which could not be defined prior to developing the protocol. If there is sufficient data for this analysis, we will analyse the clinic and patient-level factors related to the choice to prescribe and the choice of antibiotic, or AWaRe category using mixed-effect regression analysis. As outlined in the protocol we will also be evaluating guideline compliance with the WHO AWaRe Book guidance to compare observed prescribing patterns (the decision to prescribe and the choice of antibiotic, dose, duration when an antibiotic is prescribed). Data collected at the facility level on antibiotic availability and price will be incorporated into this analysis. Provided there is suitable variation in case-mix between facilities, further analyses comparing prescribing practices between clinics will be undertaken using causal variance decomposition. A more detailed statistical analysis plan will be developed prior to starting analysis. As part of a future master protocol building on this work, we will include this analysis plan and guidance defining minimum sample sizes.  

**It is unclear whether representatives from the participating clinics are invited to participate in the project's evaluation.**

 Representatives from the countries and clinics participating in the research are invited to be part of the analysis and to use their own data for their own research questions. The results from each clinic will be fed back to country leads and clinic leads in the form of PDF reports of their common presentations, antibiotic prescribing practices as well as in the form of an interactive analytical dashboard that allows partners to explore their own datasets and use it for publications, reports and more to inform local research and policy interventions. We will also conduct a feasibility survey at the end of the study with participating data collectors at clinics and country leads to gain feedback on the challenges of implementation of this study.
